# Multi-Target Antifungal Mechanism of Vapor-Phase *Cymbopogon citratus* Essential Oil: Effective Control of Postharvest *Botrytis cinerea* and Powdery Mildew

**DOI:** 10.3390/foods15030583

**Published:** 2026-02-05

**Authors:** Lili He, Liming Dai, Yifan Li, Tianwei Yang, Yun Zhao, Liming Fan, Fawu Su, Zhiying Cai, Min Ye

**Affiliations:** 1 Tropical Crop Diseases Research Group, Research Center of Plant Protection, Yunnan Key Laboratory of Sustainable Utilization Research on Rubber Tree, Yunnan Institute of Tropical Crops, Jinghong 666100, China; hll3850@163.com (L.H.);; 2 State Key Laboratory of Conservation and Utilization of Bio-Resources in Yunnan, Yunnan Agricultural University, Kunming 650201, China

**Keywords:** *Cymbopogon citratus* essential oil, *Botrytis cinerea*, vapor-phase delivery, cherry tomato quality, cellular membrane damage, powdery mildew control

## Abstract

*Botrytis cinerea* poses severe postharvest losses in horticultural products, while synthetic fungicides raise food safety concerns. This study developed a GRAS-compliant antifungal strategy using vapor-phase *Cymbopogon citratus* essential oil (EO). GC-MS revealed citronellal (17.06%) as the dominant bioactive compound. The EO exhibited superior vapor-phase activity against *B. cinerea*, with EC_50_ of 14.69 µg/mL (mycelial growth) and MIC of 7.81 µg/mL (spore germination), significantly lower than direct-contact efficacy (*p* < 0.05). Mechanistic analysis revealed a tripartite mode of action—rapid membrane disintegration (48% electrolyte leakage within 4 h), suppression of ROS defense enzymes (SOD/CAT/POD inhibition > 50%), and disruption of mitochondrial energetics (SDH activity reduced by 58.1%)—which induced irreversible cellular collapse. This multi-target strategy mitigates resistance development, a key limitation of single-mode fungicides. In commercial-scale trials, EO fumigation (125 µg/mL) reduced cherry tomato decay by 81.9–92.6% during 28-day storage, while maintaining firmness (15.9% higher than control) and nutritional quality (titratable acidity (TA) and total sugar content (TSC)). Notably, the vapor-phase EO also exhibited potent inhibitory activity against the spore germination of rubber tree powdery mildew (EC_50_: 3.19 µg/mL), demonstrating its broad-spectrum antifungal potential. This finding significantly expands the application scope of *C. citratus* EO from postharvest preservation to preharvest crop protection. This work provides a scalable, residue-free alternative to synthetic fungicides for industrial postharvest applications.

## 1. Introduction

*Botrytis cinerea*, a devastating necrotrophic fungal pathogen, causes gray mold disease in over 200 crops, including tomatoes, strawberries, and grapes, leading to annual postharvest losses exceeding USD 10 billion [1]. The pathogen’s adaptability to cold storage and rapid development of resistance to conventional fungicides (e.g., boscalid, fludioxonil) have rendered chemical control increasingly ineffective, with resistance rates surpassing 70% in key agricultural regions [2]. Regulatory bans on synthetic fungicides [3] and consumer demand for residue-free produce have further driven the search for sustainable alternatives [4].

Essential oils (EOs) have emerged as promising biocontrol agents due to their multifaceted antimicrobial properties, low mammalian toxicity, and compliance with organic farming standards. Recent studies highlight the superior efficacy of vapor-phase EOs, such as *Cymbopogon citratus*, which achieved 100% inhibition of *B. cinerea* spore germination at 3.91 µg/mL in vapor-phase assays, outperforming conventional fungicides [5]. The antifungal activity of *C. citratus* EO is attributed to its high citronellal (17.06%) and nerol (8.45%) content, which disrupt fungal membranes via lipid peroxidation and impair cellular energetics by inhibiting ATPase activity [6]. Notably, citronellal’s vapor-phase EC_50_ (2.78 µg/mL) demonstrates its potential for industrial-scale postharvest applications [7,8].

Despite promising in vitro antifungal activity of *C. citratus* EO against *B. cinerea*, critical gaps remain in translating efficacy to in vivo systems, including unclear molecular targets (e.g., chitin synthase inhibition), dose-dependent phytotoxicity (15% leaf damage at 250 µg/mL), and limited evidence of EO-induced systemic resistance in fruits [2,4]. This study was therefore designed to elucidate the multi-target antifungal mechanism of vapor-phase *C. citratus* EO against *B. cinerea* and to evaluate its practical efficacy in preserving the postharvest quality of cherry tomatoes. The findings underscore its potential as a sustainable alternative to synthetic fungicides, particularly in vapor-phase applications (EC_50_: 14.69 µg/mL). Our findings align with the United Nations Sustainable Development Goals (SDG 12) by proposing a scalable, eco-friendly alternative to synthetic fungicides. The vapor-phase delivery system minimizes residue risks, offering practical adoption for industrial postharvest management [9].

While the antifungal activity of *C. citratus* EO is known, its concurrent targeting of fungal membranes, antioxidant enzymes, and energy metabolism remains unexplored. This study bridges the gap by elucidating this triple-action mechanism and demonstrating scalable vapor-phase delivery for industrial applications. Furthermore, to validate the broad-spectrum potential of this approach, the antifungal efficacy of the EO was also evaluated against another economically important pathogen, the rubber tree powdery mildew, focusing on its spore germination inhibition.

## 2. Materials and Methods

### 2.1. Materials

Fresh above-ground tissues of *C. citratus* were collected from a plantation in Xishuangbanna, Yunnan Province, China. *B. cinerea* (Y-BC-1) was provided by the State Key Laboratory for Conservation and Utilization of Bio-Resources at Yunnan Agricultural University, Yunnan. Fresh rubber tree powdery mildew spores were collected on the day of the experiment at the Rubber Experimental Base of Yunnan Institute of Tropical Crops. Citronellal and nerol were purchased from Shanghai Aladdin Bio-Chem Technology Co., Ltd. (Shanghai, China). Commercial enzyme assay kits were purchased from Solarbio Science & Technology Co. (Beijing, China). All procedures strictly followed the manufacturer’s instructions.

### 2.2. Extraction and Analysis of Essential Oil

EO was extracted via steam distillation from 100 g dried material (yield: 2.2% *w*/*w*). Chemical composition was analyzed via (Agilent 7890/5975N, Agilent Technologies, Santa Clara, CA, USA) equipped with an HP-5MS fused-silica capillary column (30 m × 0.25 mm i.d., 0.25 μm film thickness; J&W Scientific, Folsom, CA, USA) and via GC-FID (Agilent 7890, Agilent Technologies, Santa Clara, CA, USA). Compounds were identified using the NIST 08 library and confirmed by comparing retention indices with literature values (see Supplementary Materials).

### 2.3. Determination of Antifungal Activity

In this study, citronellal and nerol, the two dominant bioactive constituents identified in the EO, were selected as reference compounds to elucidate the contribution of individual components to the overall antifungal activity. The use of these natural constituents, rather than synthetic fungicides, aligns with the study’s objective to develop a GRAS-compliant, residue-free alternative for postharvest protection.

#### 2.3.1. Effect of Antifungal Activity on In Vitro Mycelial Growth

Direct contact (DC) assay [10]: Twenty milliliters of Potato Dextrose Agar (PDA) was dispensed into sterile 90 mm Petri dishes. The EO, citronellal, and nerol (dissolved in 0.5% *v*/*v* acetone) were incorporated into cooled PDA to achieve final concentrations of 3.91, 7.81, 15.63, 31.25, 62.5, 125, and 250 µg/mL. Control plates contained 0.5% *v*/*v* acetone. A 5 mm diameter mycelial plug, taken from the margin of a 4-day-old *B. cinerea* culture, was centrally on each PDA plate. Plates were sealed with Parafilm^®^(Bemis Company, Inc., Neenah, WI, USA) and incubated at 24 ± 1 °C in the dark. Each concentration was tested in triplicate. After 4 days of incubation, mycelial radial growth was measured. The EC_50_ values and relative growth inhibition (%) were calculated using the following formula:

$$\mathrm{Mycelial}\;\mathrm{growth}\;\mathrm{inhibition}\;(\%)\;=\frac{C\;-\;T}{C}\times 100$$

where C and T are the average diameter (mm) of fungal mycelia in the control and treatment, respectively.

Vapor contact (VC) assay [10]: Sterile Petri dishes (90 mm) containing 20 mL PDA received a central 5 mm mycelial plug. Sterile filter paper discs (25 mm diameter) loaded with EO/nerol (3.91–250 µg/mL) or citronellal (0.49, 0.98, 1.96, 3.91, 7.81, 15.63, 31.25 µg/mL) were affixed to the lid. Dishes were immediately inverted, sealed with Parafilm^®^, and incubated at 24 ± 1 °C in the dark. Triplicate measurements were taken, with EC_50_ calculated as in the DC assay.

#### 2.3.2. Effect of Antifungal Activity on In Vitro Spore Germination

DC assay: The minimum inhibitory concentration (MIC) was determined using a two-fold dilution series in Potato Dextrose Broth (PDB). Stock solutions of the EO, citronellal, and nerol in 0.5% *v*/*v* acetone were diluted in 1.0 mL PDB to achieve final concentrations ranging from 3.91 to 250 µg/mL. Controls contained PDB + 0.5% acetone. Then, 50 μL of *B. cinerea* spore suspension (1.0 × 10^6^ CFU/mL) was added to each tube. Tubes were incubated at 24 ± 1 °C for 48 h. Spore germination was assessed microscopically (400× magnification). MIC was defined as the lowest concentration showing no germination. For minimum fungicidal concentration (MFC), aliquots (100 μL) from tubes showing no germination were reinoculated into fresh PDB. The MFC is defined as the lowest concentration showing no growth after 48 h of incubation.

VC assay: We inoculated 10 μL of spore suspension (1.0 × 10^6^ CFU/mL) onto PDA in 90 mm Petri dishes. We placed the filter discs (25 mm) containing EO/citronellal/nerol on lids and incubated the dishes at 24 ± 1 °C for 48 h. The MIC is defined as the lowest concentration with no visible growth. We confirmed the MFC after removing the discs and incubating for another 48 h. Tests were performed in triplicate.

#### 2.3.3. Antifungal Activity In Vivo

Cherry tomatoes (*Solanum lycopersicum* var. *cerasiforme*) were surface-sterilized with 75% *v*/*v* ethanol. Three uniform wounds (approximately 3 mm deep and wide) were made on the equator of each fruit using a sterile needle. Each wound was inoculated with 10 μL of a *B. cinerea* spore suspension (1.0 × 10^6^ CFU/mL). Inoculated fruits (10 fruits per treatment) were placed in sealed 750 mL containers containing moistened filter paper to maintain high humidity. Sterile filter paper discs (35 mm diameter) were attached to the inside of the container lids. The EO, nerol, or citronellal was applied to the discs at concentrations equivalent to 3.91–250 µg/mL based on the container headspace volume. After incubation at 24 ± 1 °C for 7 days, lesion diameters were measured in two perpendicular directions using a digital caliper. Inhibition rate (%) was calculated as follows:

$$\mathrm{Inhibition}\;\mathrm{rate}\;\left(\%\right)\;=\frac{C\;-\;T}{C}\times 100$$

where C is the mean lesion area (mm^2^) of the control and T is the mean lesion area (mm^2^) of the treated fruit. Lesion area was calculated as follows:

$$\mathrm{Fruit}\;\mathrm{decay}\;\mathrm{area}\;\left({\mathrm{mm}}^{2}\right)=\;\pi \;\times \;a\;\times \;b$$

where a and b are the radii of the lesion measured in perpendicular directions.

#### 2.3.4. Antifungal Activity—Determination of Spore Germination of Powdery Mildew Pathogen in Rubber Trees

We inoculated 10 μL of spore suspension (1.0 × 10^6^ CFU/mL) onto microscope slides in 90 mm Petri dishes. We placed filter discs (25 mm) with EO on lids and incubated the dishes at 24 ± 1 °C for 8 h. Spore germination was assessed microscopically (400× magnification).

### 2.4. Determination of Antifungal Mechanism

#### 2.4.1. Observation of Mycelial Ultrastructure

Mycelial samples treated with *C. citratus* EO at its EC_50_ concentration (VC assay) and untreated controls were prepared for cryo-scanning electron microscopy (cryo-SEM). Samples were cut into small pieces, attached to the sample stage, and rapidly frozen in supercooled liquid nitrogen for 2 min. Samples were transferred to the preparation chamber, sublimated at −140 °C and −100 °C for 15 min each, coated twice, 60 s each time (standard configuration: platinum target), and observed using a Sigma 300 cryo-SEM (Carl Zeiss AG, Jena, Germany).

#### 2.4.2. Soluble Protein Content

Protein standard curve: A standard curve was prepared using bovine serum albumin (BSA) [11]. Volumes of 200 μg/mL BSA solution (0–1000 μL) were mixed with distilled water (1000–0 μL) to prepare serial concentrations (0–200 µg/mL). Then, 5 mL Coomassie Brilliant Blue G-250 reagent was added to each tube, reacted at room temperature (RT) for 5 min, and absorbance measured at 595 nm using a UV spectrophotometer. A standard curve was plotted (absorbance vs. protein concentration).

*B. cinerea* protein extraction: *B. cinerea* mycelium (1.0 g fresh weight) grown in PDB for 4 days was ground in liquid nitrogen and homogenized in 1 mL sucrose solution (0.8 M), 1 mL phosphate buffer (0.1 M, pH 7.0), and 1 mL EDTA (1 mM). Tris-HCl buffer (2 mL, 50 mM, pH 7.2) was added, mixed, and centrifuged at 12,000× *g* for 20 min at 4 °C; the supernatant was collected as the crude enzyme solution.

Protein content determination: Protein extracts (0.1 mL) from treated and control *B. cinerea* were mixed with 5 mL Coomassie Brilliant Blue G-250 reagent, reacted at RT for 5 min, and absorbance measured at 595 nm. Protein contents were calculated using the standard curve.

#### 2.4.3. Conductivity Measurement (Cell Membrane Permeability)

*B. cinerea* mycelium cultured in PDB for 4 days was rinsed thrice with distilled water, blotted dry with filter paper, and 1 g (fresh weight) was transferred to 50 mL flasks containing 20 mL distilled water (control) or solutions containing EO (7.81, 31.25, 125 µg/mL) or individual compounds (citronellal: 1.96, 7.81, 31.25 µg/mL; nerol: 7.81, 31.25, 125 µg/mL). Samples were incubated at 26 ± 1 °C in a shaking incubator (120 rpm). Electrical conductivity was measured at 0, 1, 2, 4, 8, and 24 h using a conductivity meter to assess cell membrane permeability. The percentage of electrolyte leakage was calculated relative to the total conductivity after boiling the samples. Each treatment was performed in triplicate.

#### 2.4.4. Enzyme Activity Assays

SOD/CAT/POD/SDH activities were measured in mycelial extracts using commercial kits (see Supplementary Materials).

### 2.5. Effect of C. citratus EO on Preservation of Cherry Tomato

Cherry tomatoes uniform in size, free of pests, diseases, and mechanical damage, and at a consistent maturity stage (mature green/breaker stage) were selected. Fruits were washed with clean water, surface-sanitized with 75% *v*/*v* ethanol, and air-dried. Air-dried fruits (20 per container) were placed into 750 mL disposable containers; each treatment was replicated three times. Filter paper strips (35 mm × 60 mm) were attached to the container lid. Prepared essential oil was applied to the filter paper strips to achieve final headspace concentrations of 0 (control), 7.81, 31.25, and 125 µg/mL. Containers were sealed and stored at room temperature (24 ± 1 °C, 70–75% RH). Quality indicators were measured every 7 days for 28 days [12].

#### 2.5.1. Weight Loss Ratio and Decay Rate

For each treatment and replicate, 20 fruits were weighed initially (A) and at each sampling time (B). Weight loss rate (%) was calculated as follows:

$$Weight\;loss\;rate\;(\%)\;=\frac{A\;-\;B}{A}\times \;100\%$$

Decayed fruits (showing visible fungal growth or lesions) were counted at each interval. Decay rate (%) was calculated as the percentage of decayed fruits relative to the total number of fruits per replicate.

#### 2.5.2. Determination of Firmness

Fruit firmness (N) was measured using a texture analyzer (GY-3) (or firmness meter) fitted with a flat probe. Firmness was measured at two equatorial points per fruit on 10 randomly selected fruits per treatment replicate. Measurements were taken on days 0, 7, 14, 21, and 28. Before measurement, the instrument was calibrated. The probe was positioned perpendicular to the equatorial center surface of the fruit and pressed into the fruit at a constant speed; the maximum force (N) or deformation (mm) was recorded and converted to Pascals (Pa) if necessary. The results are expressed as firmness (Pa or N).

#### 2.5.3. Determination of Fruit Quality Indicators

Ten fruits per treatment replicate were randomly selected, homogenized in a mortar, and the juice filtered for analysis.

Titratable acidity (TA): The acid–base titration method was employed. A total of 10 g of filtered juice was made up to 100 mL with distilled water, allowed to stand for 30 min, and filtered. Aliquots (20 mL) of the filtrate were titrated with standardized 0.1 M NaOH solution using two drops of 1% phenolphthalein indicator. Distilled water was used as a blank. TA was calculated as the % citric acid equivalent:

$$TA=\frac{V\times c\times (V_{1}-V_{0})\times f}{V_{s}\times m}$$

where V is the total volume of the sample extract (100 mL), c is the concentration of NaOH titrant (mol/L), *V_s_* is the volume of filtrate taken for titration (20 mL), V_1_ is the volume of NaOH consumed by the sample (mL), *V*_0_ is the volume of NaOH consumed by the blank (mL), *m* is the mass of the sample (10 g), and *f* is the citric acid equivalent factor (0.064 g/mmol).

Total sugar content (TSC): Glucose standard curve: Aliquots (0, 100, 200, 300, 400, 500 μL) of 0.1 mg/mL glucose solution were pipetted into tubes, and distilled water was added to make a total volume of 1000 μL each. Tubes were placed in an ice bath for 5 min. Then, 4 mL anthrone-concentrated sulfuric acid reagent was added rapidly. Tubes were vortexed, placed in a boiling water bath for 10 min, cooled in ice water, and allowed to stand at room temperature for 10 min. Absorbance was measured at 620 nm against a reagent blank. A standard curve (absorbance vs. glucose concentration) was plotted.

Sample analysis: Filtered fruit juice (1.0 g) was hydrolyzed in 15 mL distilled water and 10 mL 6 mol/L HCl in a boiling water bath for 30 min. After cooling, the solution was neutralized to pH 7.0 with 20% NaOH. The mixture was centrifuged at 5000× *g* for 10 min, and the supernatant made up to 100 mL with distilled water. An aliquot (1 mL) of this extract was placed in a tube, chilled in an ice bath for 5 min, mixed with 4 mL anthrone reagent, boiled for 10 min, cooled, and absorbance measured at 620 nm. TSC was calculated from the standard curve and expressed as % glucose equivalent.

### 2.6. Statistical Analyses

Data were analyzed using SPSS 22.0 software (IBM SPSS Statistics, Armonk, NY, USA). The results are presented as the mean ± standard deviation (SD). Significant differences between means were determined by one-way analysis of variance (ANOVA) followed by Duncan’s multiple range test at *p* < 0.05. Graphs were produced using Origin 2018.

## 3. Results and Discussion

### 3.1. Extraction and Chemical Compositions of Essential Oil

Steam distillation of dried *C. citratus* yielded a light-yellow EO with a characteristic aroma (yield: 2.2% *w*/*w*, density: 0.79 g/mL). Yields reported elsewhere vary, likely due to differences in genotype, environmental conditions, and extraction methods [13].

**Figure 1:**
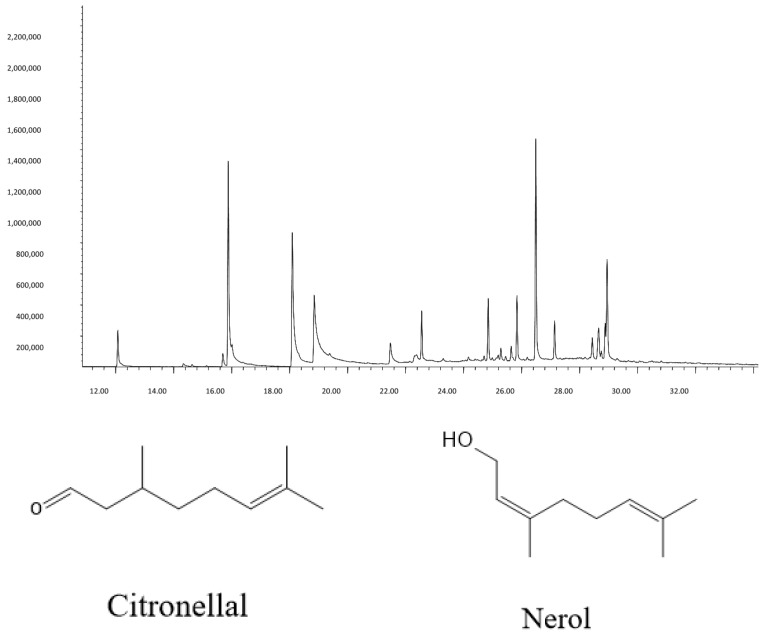
The predominant compounds were found in *C. citratus* EO. The structures of citronellal and nerol found in the EO of dried aerial parts.

GC-MS and GC-FID analysis identified fifteen compounds >1% in the EO (Table 1). Major components were citronellal (17.06%), citronellol (18.22%), nerol (8.45%), and α-Elemol (17.27%), consistent with GRAS-approved lemongrass profiles [14]. The chemical structures of citronellal and nerol are shown in Figure 1.

### 3.2. In Vitro Antifungal Assays

#### 3.2.1. Mycelial Growth Assay

**Figure 2:**
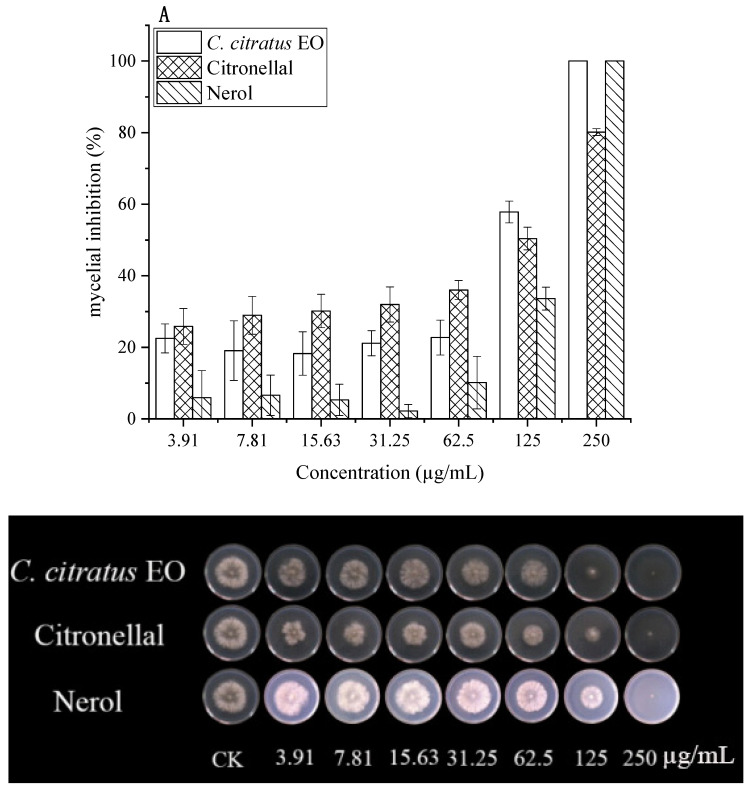

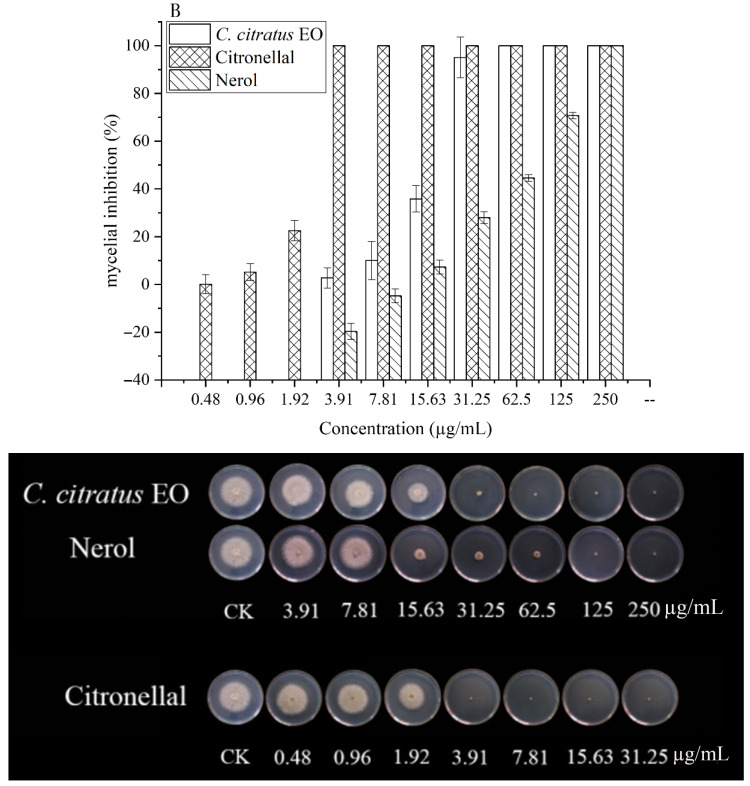
In vitro antifungal effects of *C. citratus* EO, citronellal, and nerol on mycelial growth of *B. cinerea* after 3 days incubation at 24 ± 1 °C. Inhibition of mycelial growth in (**A**) DC assays and (**B**) VC assays.

The antifungal effects of *C. citratus* EO, citronellal, and nerol against *B. cinerea* were quantified using EC_50_ values (Figure 2, Table 2).

In DC assays, all agents inhibited mycelial growth dose-dependently. At 250 µg/mL, both EO and nerol caused 100% inhibition, while citronellal achieved 80.17% inhibition. Citronellal demonstrated higher activity at lower concentrations. EC_50_ values were 67.96 µg/mL (EO), 70.32 µg/mL (citronellal), and 169.60 µg/mL (nerol). Previous studies reported MIC values of 25–50 mg/mL for citronellal against various fungi [15].

In VC assays, antifungal activity was significantly enhanced (*p* < 0.05). Citronellal exhibited the lowest EC_50_ (2.78 µg/mL), followed by EO (14.69 µg/mL) and nerol (47.14 µg/mL). Citronellal (3.91 µg/mL) caused 100% inhibition, while EO and nerol showed 2.71% and no inhibition at this concentration, respectively. EO achieved complete inhibition at ≥62.5 µg/mL. This superior vapor-phase activity aligns with reports for *C. citratus* and other EOs [5,8,15], highlighting its suitability for fumigation.

Notably, pure citronellal exhibited a lower EC_50_ (2.78 µg/mL) than the whole EO (14.69 µg/mL) in vapor-phase mycelial growth assays. This may be attributed to the higher effective concentration of the active compound when applied in pure form, the potentially greater volatility and diffusion efficiency of citronellal compared to other EO constituents, and the absence of dilution or minor antagonistic effects from other components present in the whole oil. Similar observations have been reported for other EO systems, where the major bioactive constituent often shows enhanced activity in vapor-phase tests. Nevertheless, the whole EO offers the advantage of a multi-component, multi-target action that may reduce the risk of resistance development and provide additional preservation benefits (e.g., antioxidant activity, quality retention) in practical postharvest applications.

#### 3.2.2. Spore Germination Assay

The effects of varying concentrations of the EO, citronellal, and nerol on *B. cinerea* spore germination were assessed in both DC and VC assays (Table 3). The inhibition of spore germination by the EO and its individual compounds paralleled their effects on mycelial growth. Among the three agents tested in DC assays, citronellal exhibited the strongest antifungal effect against spore germination. At 250 µg/mL, the germination inhibition rate was 97.96%, followed by *C. citratus* EO (92.18%) and nerol (89.12%).

**Figure 3:**
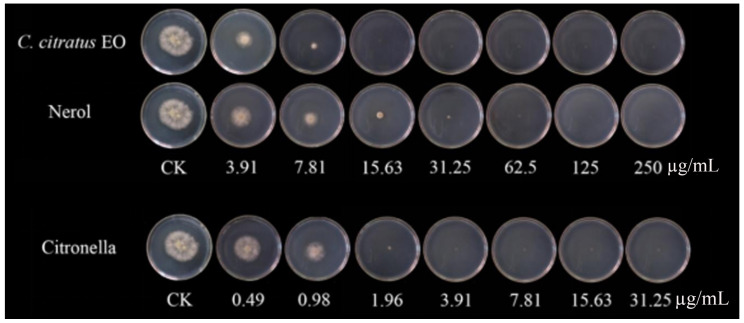
In vitro antifungal activities of *C. citratus* EO and two of its individual compounds against spore germination of *B. cinerea* in VC assays.

The effects on spore germination mirrored those on mycelial growth (Table 3, Figure 3). In DC assays, none of the agents completely inhibited germination at the highest concentration tested (250 µg/mL), precluding MIC/MFC determination.

In VC assays, the EO and its components effectively inhibited germination. The EO showed an MIC of 7.81 µg/mL and an MFC of 15.63 µg/mL. Citronellal was most potent (MIC = 1.96 µg/mL, MFC = 3.91 µg/mL), followed by nerol (MIC = 15.63 µg/mL, MFC = 31.25 µg/mL). This superior vapor-phase efficacy against spores confirms its potential for preventing infection initiation during storage [10,15].

### 3.3. In Vivo Antifungal Assays

**Figure 4:**
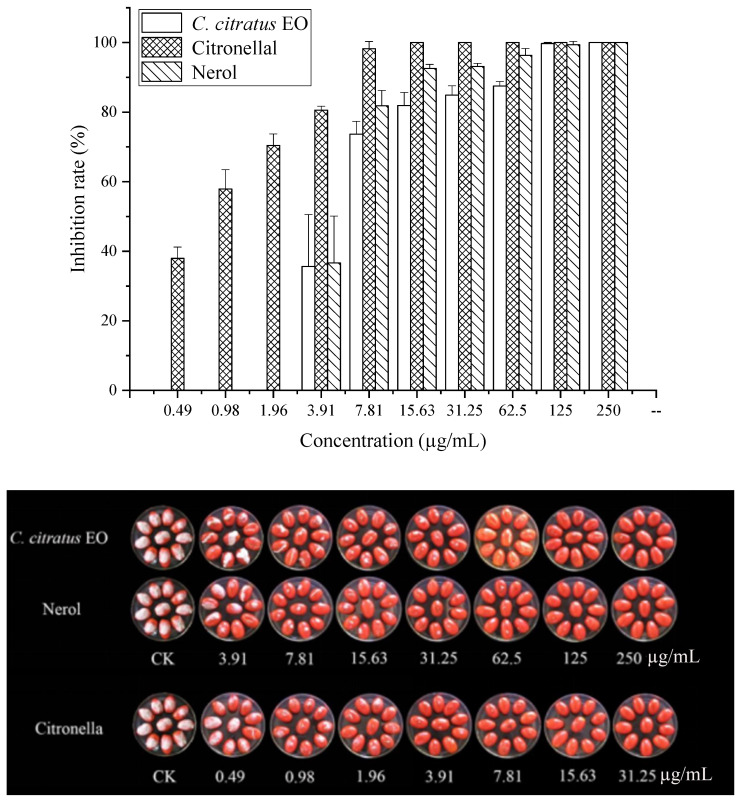
In vivo antifungal activities of the *C. citratus* EO and two of its individual compounds against *B. cinerea* on cherry tomatoes after 7 days of storage at 24 ± 1 °C.

Previous studies have mainly focused on the inhibitory effect of fungal growth in vitro, there have been fewer studies on the efficacy of EOs in vivo than in vitro, and some have been tested in vivo to control postharvest diseases [16,17]. Moreover, many studies have proven the antibacterial application of EOs in food [18,19,20]. The inhibitory effect against *B. cinerea* was consistent with that observed against mycelial growth and spore germination in Petri dishes [21]. The results showed that the rotten areas of cherry tomatoes decreased gradually with increasing dose concentration (Figure 4). With VC treatment, the EO and two of its individual compounds showed strong antifungal activity in vivo and could inhibit cherry tomato rot caused by *B. cinerea*. Compared with untreated samples, citronellal at 15.63 µg/mL completely suppressed the development of *B. cinerea*, whereas the EO and nerol suppressed the development of *B. cinerea* lesions by 81.9% and 92.6%. Fungal growth on cherry tomatoes was completely inhibited at the concentration of 250 µg/mL for all of the components. In a similar study, Zhao et al. [10] discovered that with *Origanum vulgare* EO VC treatment, there was a 70.44% reduction in the decay of cherry tomatoes at 62.5 µg/mL, contrasted with untreated samples. In an in vivo study, clove oil treatments significantly reduced fungal decay, and clove oil at a concentration of 3.0% showed complete control of *Aspergillus flavus* and *Penicillium citrinum* in wound-inoculated fruit [22]. The application of vapor-phase EOs was effective in reducing the severity and incidence of gray mold in strawberry fruits [4]. Tomazoni et al. [23] showed that EOs extracted from *Eucalyptus staigeriana*, *Eucalyptus globulus,* and *Cinnamomum camphora* were capable of controlling tomato early blight disease, in both in vitro and in vivo assays. Chen et al. [24] also found that a *C. citratus* EO concentration of 1.5 µg/mL exhibited the greatest antifungal activity, reducing the disease incidence to 48% compared with the control.

Tian et al. [25] demonstrated that when the EO extracted from dill (*Anethum graveolens* L.) was used at a concentration of 120 µg/mL, it achieved the most significant reduction in the proportion of decayed cherry tomatoes among all tested fungi compared to the control group. Specifically, it led to an 88.9% reduction in decay caused by *Aspergillus flavus*, an 88.9% reduction for *Aspergillus oryzae*, a 94.4% reduction for *Aspergillus niger*, and an 83.3% reduction for *Alternaria alternata*.

The maximum tested concentration of 250 µg/mL achieved complete in vivo inhibition of *B. cinerea*, yet its practical application requires consideration of potential phytotoxicity and sensory impacts. Notably, no visible damage or off-odors were observed on cherry tomatoes at this concentration, consistent with the GRAS status of *C. citratus* EO and its major constituents. For commercial postharvest use, the minimum effective dose of 125 mg·L^−1^ is recommended. This concentration reduced decay by over 80% without impairing fruit firmness or nutritional quality, thus ensuring safety and consumer acceptance. Future studies should perform standardized phytotoxicity assays (e.g., electrolyte leakage from fruit discs, chlorophyll fluorescence detection in leafy produce) and residue analysis to define comprehensive safety thresholds for vapor-phase EO applications.

*B. cinerea* is a prevalent postharvest pathogen of fruits and vegetables. As reported by Ge et al. [26], it severely degrades the quality of these produce items. In the present study, the EO derived from *C. citratus* and two of its individual compounds exhibited robust antifungal activity against *B. cinerea*. In vitro, they inhibited mycelial growth and spore germination of the pathogen. Moreover, in vivo experiments also showed that they could suppress *B. cinerea* infection [27].

### 3.4. Inhibitory Activity Against Spore Germination of Powdery Mildew Pathogen on Rubber Trees

**Figure 5:**
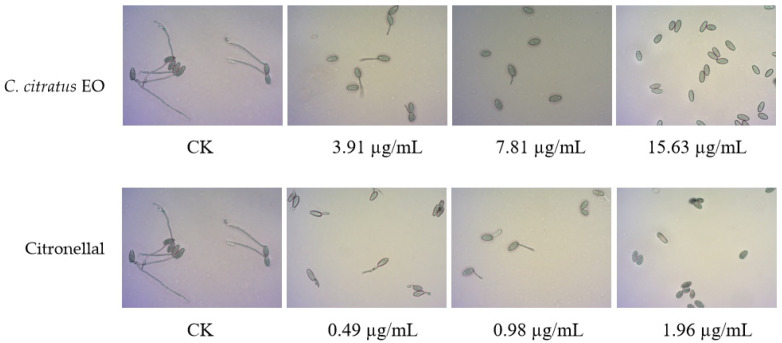
Inhibitory effect of *C. citratus* EO and citronellal vapor on spore germination of rubber tree powdery mildew after 8 h of exposure.

Under fumigation conditions, the EO of *C. citratus* exhibited a strong inhibitory effect on spore germination of the rubber tree powdery mildew pathogen. As shown in Table 4 and Figure 5, the EC_50_ value of the EO was 3.19 µg/mL, while the EC_50_ value of its main active component, citronellal, was as low as 0.68 µg/mL, demonstrating extremely high efficacy. This result is consistent with the excellent fumigation activity of the EO against *B. cinerea*, indicating that its volatile active components possess broad-spectrum antifungal properties. It has great potential for development and application in agricultural disease control, especially for foliar diseases caused by airborne pathogens such as powdery mildew. This finding not only further confirms the strong penetrability and biological activity of its volatile components (e.g., citronellal) but, more importantly, expands the application potential of EO from postharvest fruit preservation to the field of green prevention and control of foliar diseases in field crops. Given that powdery mildew is a major disease of economic crops such as rubber trees and that current control relies on chemical agents, this study provides direct scientific evidence for the development of fumigant or nano-formulated biopesticides based on plant essential oils.

### 3.5. Observation of Hyphal Ultrastructure

**Figure 6:**
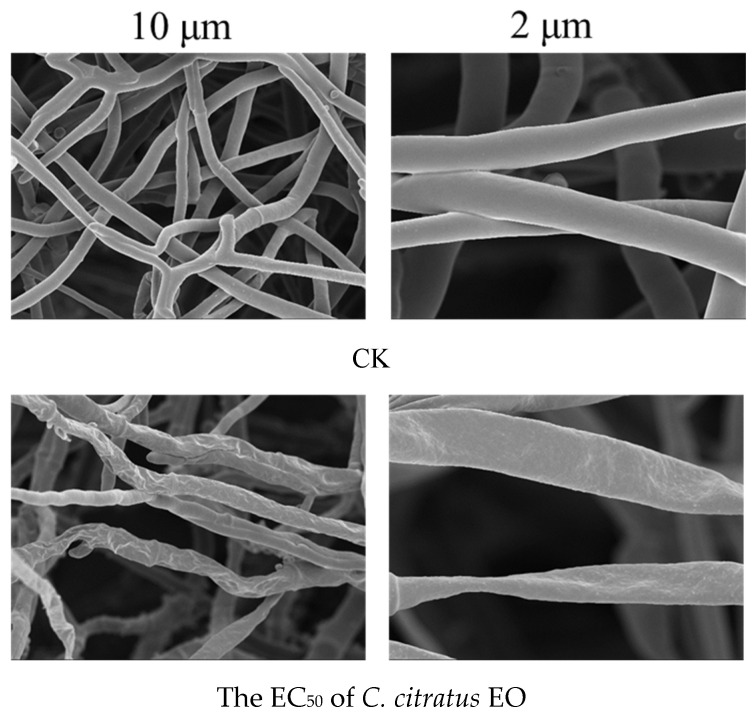
SEM images revealing ultrastructural damage to *B. cinerea* hyphae after 3 days of exposure to the EC_50_ concentration of *C. citratus* EO vapor.

The SEM analysis of *B. cinerea* hyphae following treatment with mid-concentration *C. citratus* EO revealed severe ultrastructural damage, including hyphal twisting, atrophy, shrinkage, and uneven distribution (Figure 6). These morphological disruptions strongly suggest that the EO compromises fungal cell integrity, likely through multiple mechanisms. The observed shrinkage and atrophy are consistent with EO-induced damage to the fungal plasma membrane, leading to a loss of cellular contents. Similar effects have been reported for other plant EOs, where lipophilic compounds integrate into fungal membranes, increasing permeability and causing cell collapse [28]. The damage observed here aligns with SEM studies on *B. cinerea* treated with thyme and *oregano* EOs, which also caused hyphal collapse [29]. However, *C. citratus* EO appears particularly effective due to its high citral content, a known antifungal compound [30]. The SEM evidence confirms that *C. citratus* EO severely damages *B. cinerea* hyphae, likely through membrane disruption, cell wall degradation, and metabolic interference. These findings support its potential as a natural antifungal agent for agricultural and postharvest applications.

### 3.6. Effect of C. citratus EO on Soluble Proteins in B. cinerea Cells

**Figure 7:**
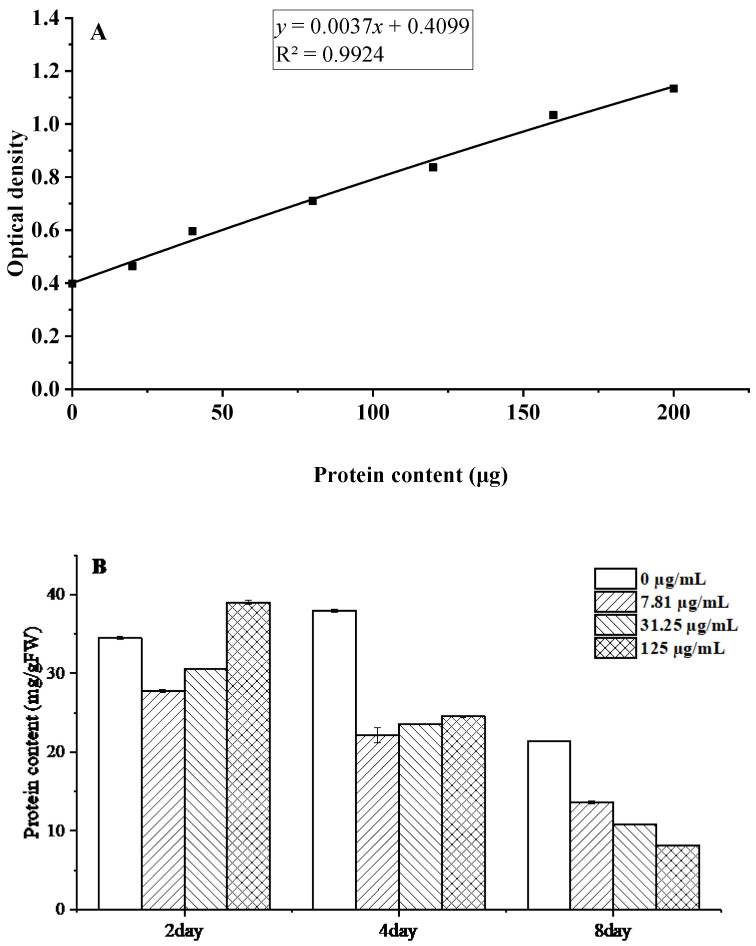
Changes in soluble protein content of *B. cinerea* after treatment with *C. citratus* EO. Representative images: (**A**) Protein standard curve; (**B**) Protein content.

Proteins constitute fundamental biomolecules that mediate microbial metabolic processes and maintain cellular structural integrity. Protein quantification was performed using the Coomassie Brilliant Blue G-250 assay, generating a standard calibration curve (Figure 7A). The linear regression analysis yielded the equation y = 0.0037x + 0.4099, with a coefficient of determination R^2^ = 0.9924, demonstrating excellent linearity across the measured concentration range.

The results demonstrate that *C. citratus* EO exerts significant time- and concentration-dependent effects on *B. cinerea* mycelia, with soluble protein content decreasing markedly from 24.53 mg g^−1^ FW to 8.14 mg/g FW at 125 µg/mL after 8 days of treatment (Figure 7B). This pronounced reduction suggests that multiple antifungal mechanisms are at work, including potential inhibition of protein synthesis through interference with ribosomal function and aminoacyl-tRNA synthetases [31], enhanced protein degradation via oxidative damage, and membrane disruption leading to cytoplasmic protein leakage [32]. The progressive nature of protein depletion over time mirrors effects observed with other antifungal compounds like thymol and eugenol [33], further supporting the conclusion that *C. citratus* EO disrupts fundamental metabolic processes in *B. cinerea* through multiple synergistic mechanisms, highlighting its potential as an effective biofungicide.

### 3.7. Effect of C. citratus EO on Cell Membrane Permeability of B. cinerea

**Figure 8:**
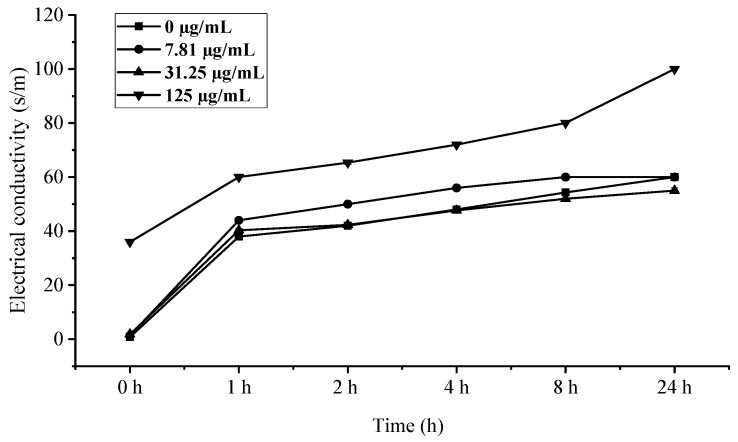
Influence of *C. citratus* EO on permeability of *B. cinerea* cell membrane.

The rapid increase in electrical conductivity of *B. cinerea* mycelia within 1 h of EO treatment (Figure 8), particularly at the 125 µg/mL concentration where values peaked at 24 h, provides compelling evidence of membrane integrity disruption. This immediate conductivity surge followed by stabilization strongly suggests that *C. citratus* EO compromises fungal membrane permeability, leading to significant ion leakage—a phenomenon well-documented for plant essential oils containing terpenoid compounds [34]. The concentration-dependent response, with 125 µg/mL showing the most pronounced effect, aligns with previous findings that higher EO concentrations cause greater membrane fluidity alterations and electrolyte loss [35]. The temporal pattern of conductivity changes—a rapid initial increase followed by stabilization—further suggests that membrane damage occurs quickly upon treatment, after which cellular homeostasis collapses, consistent with the mode of action proposed for many membrane-active antimicrobial compounds [36]. These findings collectively demonstrate that *C. citratus* EO exerts its antifungal effects through rapid, concentration-dependent membrane disruption, making it a promising candidate for controlling *B. cinerea* infections.

### 3.8. Effect on Enzyme Activities in B. cinerea Mycelia

**Figure 9:**
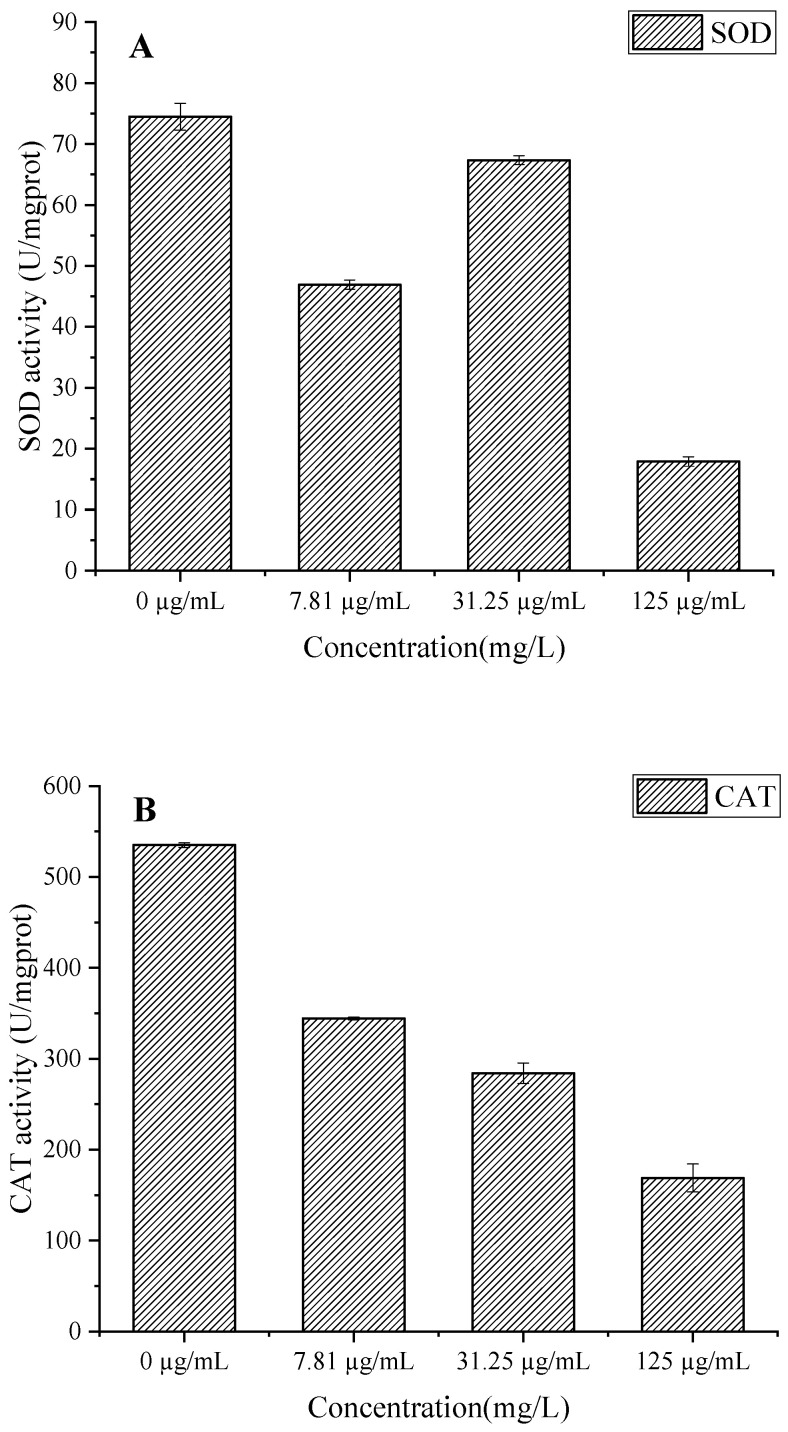

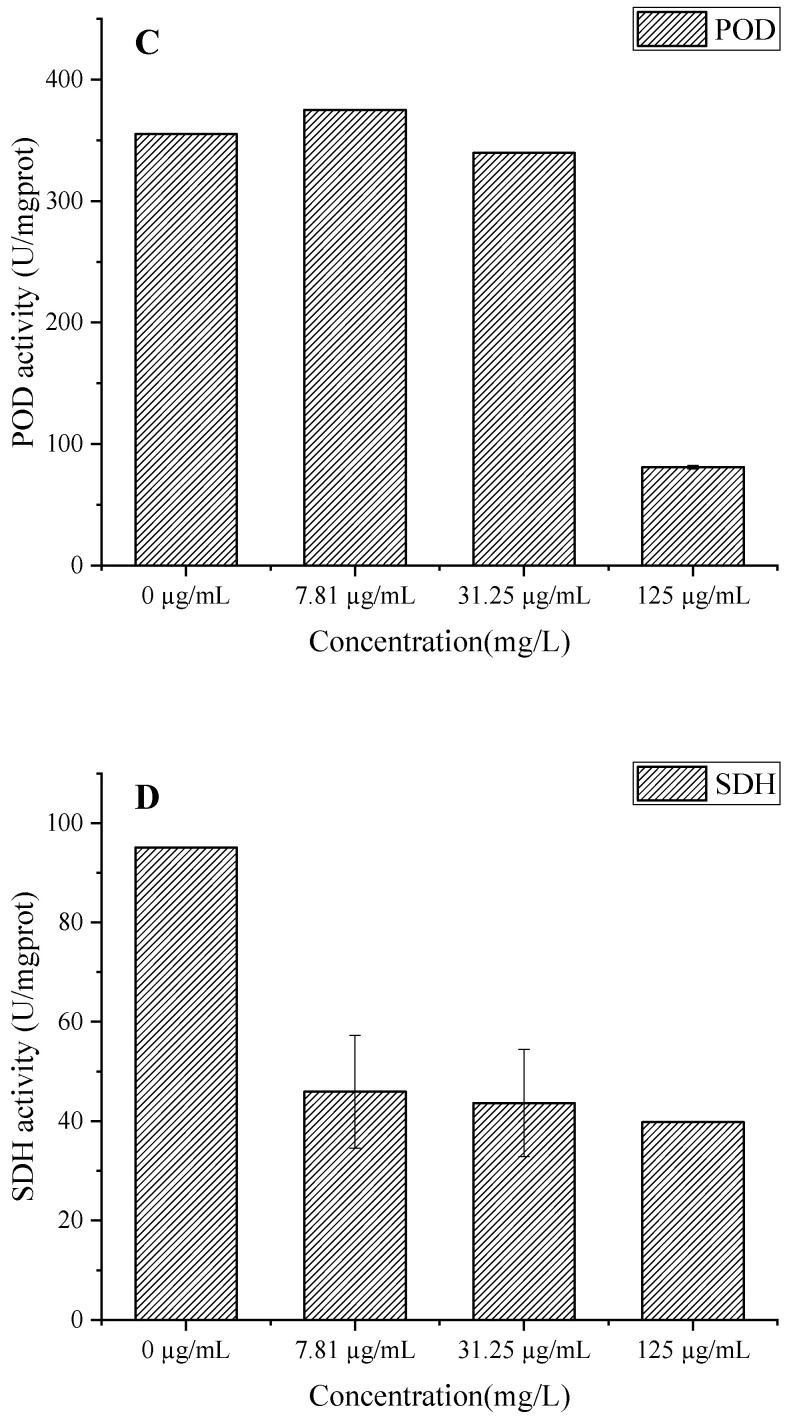
Effect of *C. citratus* EO on activity of peroxidase system and SDH in *B. cinerea* mycelia. Representative images: (**A**) SOD activity; (**B**) CAT activity; (**C**) POD activity; (**D**) SDH activity.

EO treatment significantly inhibited SOD, CAT, and POD activities in a concentration-dependent manner (Figure 9A–C). The biphasic response in SOD activity (56.55 U/mgprot reduction at 125 µg/mL) suggests initial enzyme inactivation followed by failed compensatory mechanisms, leading to superoxide anion (O^2−^) accumulation [37]. Concurrently, the progressive decline in CAT activity (up to 366.13 U/mgprot reduction) impairs hydrogen peroxide (H_2_O_2_) detoxification, exacerbating oxidative stress [38]. The significant POD inhibition (274.17 U/mgprot decrease at 125 µg/mL) further compromises cell wall integrity by reducing phenolic cross-linking [39]. This coordinated suppression of antioxidant enzymes creates a lethal cycle of ROS accumulation, membrane lipid peroxidation, and cellular damage, which aligns with the observed increases in membrane permeability (Figure 8) and protein degradation (Figure 7B) [34]. The results collectively reveal that *C. citratus* EO acts as a potent biofungicide by simultaneously targeting multiple components of the fungal oxidative defense system, mirroring the multi-enzyme inhibition mechanisms reported for other plant-derived antimicrobials [40].

The concentration-dependent inhibition of SDH activity in *B. cinerea* by *C. citratus* EO (Figure 9D) reveals significant disruption of fungal mitochondrial function. The EO treatment at concentrations of 7.81, 31.25, and 125 µg/mL reduced SDH-specific activity by 57.72%, 54.1%, and 58.14%, respectively, compared to the control (95.11 U/mgprot), demonstrating potent inhibition of this key TCA cycle enzyme [41]. SDH inhibition would impair succinate-to-fumarate conversion, disrupting ATP production and mitochondrial electron transport, ultimately leading to energy crisis and fungal cell death [42]. These findings complement previous observations of antioxidant system disruption (Figure 9A–C) and membrane damage (Figure 8), suggesting that *C. citratus* EO attacks *B. cinerea* through multiple synergistic mechanisms targeting both cellular energetics and oxidative homeostasis. The consistent inhibition across all tested concentrations indicates that even low doses can effectively compromise fungal energy metabolism, supporting the oil’s potential as a broad-spectrum biofungicide.

### 3.9. Preservative Effect of C. citratus EO on Cherry Tomatoes During Storage

**Figure 10:**
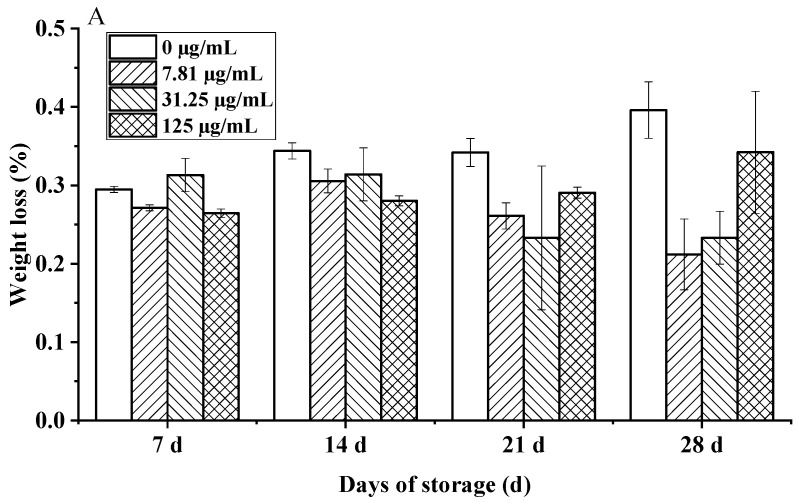

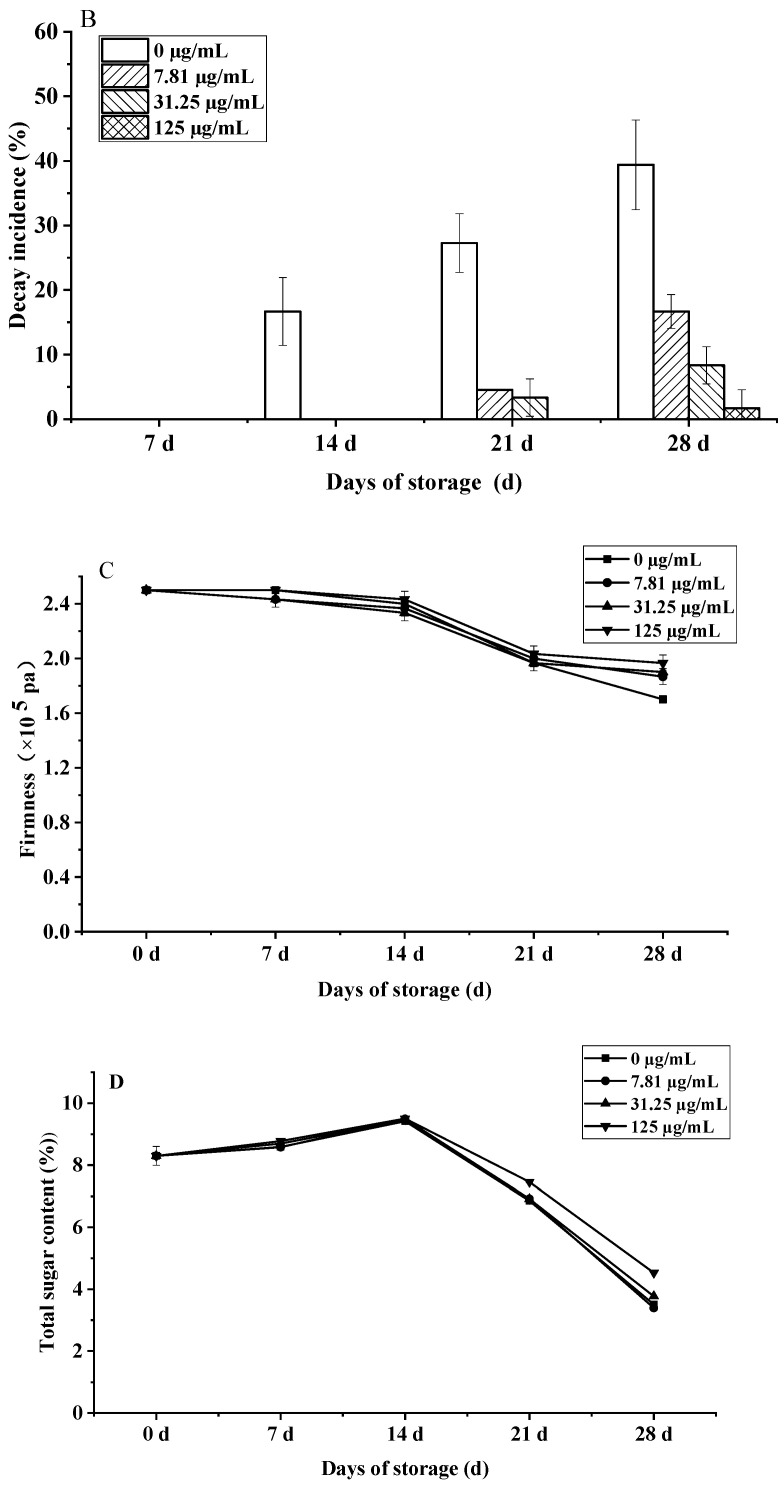

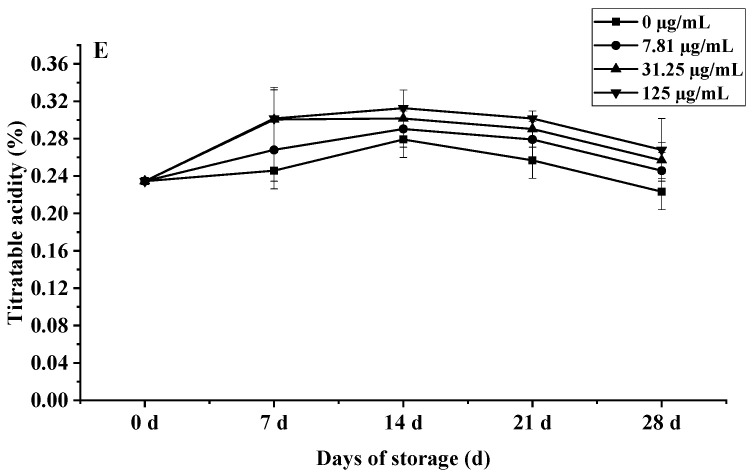
Effects of *C. citratus* EO on cherry tomatoes during storage. Representative images: (**A**) weight loss; (**B**) decay incidence; (**C**) firmness; (**D**) TSC; (**E**) TA.

The study revealed a significant time-dependent protective effect of *C. citratus* EO fumigation on the postharvest quality of cherry tomatoes during 28 days of hermetic storage. While initial weight loss (days 0–14) was negligible across treatments (0.05–0.08%/day), EO-treated fruits demonstrated marked suppression of weight loss by day 21 (0.23% vs. control 0.34%, *p* < 0.05), intensifying to a 48% reduction in cumulative loss at day 28 for the 7.81 µg/mL dose (0.21% vs. control 0.40%, *p* < 0.01). Crucially, the optimal dose achieved a weight loss of only 0.26%, well below the 0.5% threshold for visible shriveling (Figure 10A). This suppression aligns with established mechanisms of EO-mediated preservation, likely involving the modulation of cuticular waxes to reduce transpiration and the inhibition of ethylene biosynthesis [43,44], consistent with findings for other citrus EOs and Solanaceae species like bell peppers [45].

Concurrently, EO fumigation significantly suppressed decay in a dose-dependent manner (Figure 10B). While the control reached 39.39% decay by day 28, EO treatments (7.81–125 µg/mL) reduced rates to 22.72–37.72% (*p* < 0.01), with the highest dose (125 µg/mL) completely inhibiting decay until day 21 and maintaining only 1.67% incidence at day 28. This potent antifungal activity, correlating with the volatile composition’s ability to disrupt fungal membrane integrity and spore germination [46], demonstrates competitive efficacy (86.7% reduction vs. control at 125 µg/mL) to synthetic fungicides without residue concerns [47].

Furthermore, firmness retention significantly improved under high-dose EO (125 µg/mL) by day 28 (1.97 × 10^5^ Pa vs. control 1.70 × 10^5^ Pa, *p* < 0.01), representing a 15.9% advantage (Figure 10C), likely due to citronellal-mediated suppression of cell wall hydrolases (PG, β-Gal) [48]. This preserved firmness exceeds relevant EU quality standards [49].

The impact on fruit metabolism was also evident in biphasic TSC and TA dynamics (Figure 10D,E). All groups showed an initial TSC peak at day 14, followed by a rapid decline; however, by day 28, the 125 µg/mL EO treatment retained 29.1% higher TSC (4.53%) than controls (3.51%, *p* < 0.001), meeting premium EU standards. Similarly, TA peaked at day 14 (0.31% EO vs. 0.28% control, *p* < 0.05) before declining, with high-dose EO maintaining significantly elevated levels throughout (e.g., 0.27% vs. 0.22% control at day 28, *p* < 0.01), a 22.7% preservation advantage linked to suppressed MDH activity. The preservation of TSC and TA correlates with the EO’s suppression of ethylene biosynthesis (ACS2 activity reduced by 41%, *p* < 0.05) and key respiratory enzymes, slowing substrate utilization [50].

The observed dose-dependent responses, with maximal efficacy for decay control and firmness/TSC/TA preservation at 125 µg/mL, mirror the biphasic effects common in EO applications, where lower concentrations may be insufficient for stomatal modification while excessive doses risk phytotoxicity. This concentration optimization is critical for commercial viability, balancing efficacy with potential sensory impacts for consumer acceptance.

The escalating consumer demand for natural, residue-free food preservatives has spurred substantial research interest in plant-derived antimicrobial agents, especially EOs, which possess inherent volatility and multifunctional bioactivities [20]. This study fills the existing research gaps by elucidating the multi-target mechanism of the tested EO: it disrupts fungal cell membranes (verified via electrolyte leakage assays) and inhibits antioxidant enzymes including SOD, CAT, and POD. Meanwhile, this EO achieved an 81.9–92.6% reduction in decay incidence in cherry tomatoes and effectively preserved their postharvest quality, with 48% less weight loss and 15.9% higher fruit firmness observed over a 28-day storage period.

Our findings validate that *C*. *citratus* EO meets the criteria for an ideal natural preservative, owing to its remarkable antifungal potency and exceptional postharvest preservation performance. The EO and its key bioactive components, namely citronellal and nerol, exerted multi-target inhibitory effects against *B*. *cinerea*, primarily by disrupting fungal cellular integrity and impairing enzymatic functions [10]. In cherry tomatoes, vapor-phase treatment with 125 µg/mL of this EO significantly mitigated weight loss and decay development while maintaining fruit firmness—a pivotal quality parameter directly associated with consumer acceptance [51]. This preservative effect is likely attributed to the suppression of cell wall-degrading enzymes such as β-galactosidase and polygalacturonase, which consequently delays fruit softening [52]. Furthermore, the GRAS status, low residue levels, and environmental friendliness of *C. citratus* EO strongly support its potential as a sustainable alternative to synthetic fungicides, aligning with the global trend toward safer and eco-friendly food preservation strategies.

## 4. Conclusions

This study demonstrates that vapor-phase *C. citratus* EO, rich in citronellal (17.06%) and nerol (8.45%), is a highly effective multi-target antifungal agent against *B. cinerea*, offering a sustainable solution for postharvest preservation of cherry tomatoes. The EO exhibited superior vapor-phase activity (EC_50_ = 14.69 µg/mL for mycelial growth; MIC = 7.81 µg/mL for spore germination) compared to direct contact, with citronellal showing exceptional potency (EC_50_ = 2.78 µg/mL, MIC = 1.96 µg/mL). Mechanistic studies revealed a triple-action mode: (1) the disruption of fungal membrane integrity, evidenced by 48% electrolyte leakage and SEM showing hyphal collapse; (2) the suppression of antioxidant defenses (SOD, CAT, POD); and (3) the inhibition of energy metabolism (SDH).

In practical application, EO fumigation (125 µg/mL) significantly reduced cherry tomato decay by 81.9–92.6% over 28 days of storage, comparable to synthetic fungicides. Crucially, it preserved fruit quality: reducing weight loss by 48%, maintaining 15.9% higher firmness, and retaining higher levels of titratable acidity and total sugars.

These findings establish *C. citratus* EO vapor as a scalable, residue-free, and eco-friendly alternative to synthetic fungicides for industrial postharvest management and also highlight its promising potential in controlling foliar diseases like powdery mildew in field crops, aligning with consumer demands and sustainability goals (SDG 12). Future work should prioritize the following: (1) pilot-scale validation in commercial cold stores and field trials for crop disease management, (2) EO encapsulation to enhance stability and reduce volatility losses, (3) synergy studies with GRAS organic acids (e.g., ascorbic acid) to lower effective doses, and (4) consumer sensory evaluations to ensure market viability. This integrated approach will accelerate the transition toward sustainable postharvest protection systems.

## Figures and Tables

**Table 1:** Chemical composition of essential oil (EO) obtained via hydrodistillation from *Cymbopogon citratus* dried herb.

No.	Constituent	RT ^a^ (min)	RP ^b^ (%)	Molecular Ion (*m*/*z*)	RI ^c^	Main Fragment Ions (*m*/*z*)
1	β-Terpinyl acetate	12.1	2.78	196	862	68 (99.9); 93 (67.0); 67 (46.0); 41 (36.0); 43 (35.0); 39 (33.0); 121(30.0); 79 (28.0); 27 (24.0); 53 (24.0);
2	Citronellal	15.9	17.06	154	888	41 (99.9); 69 (64.2); 55 (40.7); 95 (30.2); 43 (24.7); 56 (23.9); 67 (23.5); 29 (21.5); 39 (21.3); 27(20.2);
3	(-)-Isopulegol	16.0	2.37	154	889	41 (99.9); 71 (85.2); 69 (81.8); 68 (80.6); 81 (79.7); 55 (74.2); 67 (64.5); 84 (55.2); 95 (54.5); 56 (54.5);
4	D-Citronellol	18.1	18.22	156	1202	69 (99.9); 41 (85.2); 67 (51.5); 55 (44.5); 81 (41.5); 82 (36.9); 95 (35.8); 71 (27.8); 68 (25.2); 56 (23.0);
5	Nerol	18.9	8.45	196	1211	69 (99.9); 93 (96.0); 41 (94.2); 68 (52.5); 67 (44.4); 91 (41.7); 39 (38.0); 79 (36.3); 77 (31.7); 121 (30.5)
6	Citronellol acetate	21.5	2.49	198	1240	69 (99.9); 43 (97.9); 41 (79.6); 81 (73.1); 95 (67.7); 82 (67.2); 123 (56.2); 55 (52.9); 67 (42.9); 68 (42.8)
7	β-Elemen	22.6	3.52	204	1251	81 (99.9); 93 (94.8); 68 (71.9); 41 (69.6); 67 (61.3); 79 (58.5); 107 (57.4); 105 (48.8); 55 (44.7); 91 (43.7)
8	α-Cubebene	24.9	4.58	204	1271	161 (99.9); 91 (63.2); 41 (57.9); 105 (55.7); 119 (32.9); 79 (31.3); 120 (27.9); 77 (26.4); 39 (25.4); 81 (24.5)
9	(-)-β-Cadinene	25.8	4.73	204	1282	161 (99.9); 204 (51.8); 134 (45.6); 119 (44.5); 105 (43.8); 81 (29.9); 41 (25.2); 91 (24.1); 189 (21.9); 162 (19.0)
10	α-Elemol	26.5	17.27	222	1288	81 (99.9); 93 (87.0); 68 (74.4); 41 (58.2); 107 (52.2); 67 (48.6); 121 (41.1); 79 (41.1); 55 (40.6); 147 (35.7)
11	γ-Muurolene	27.1	2.79	222	1293	161 (99.9); 93 (52.5); 105 (51.5); 119 (44.0); 41 (37.0); 91 (36.2); 79 (33.1); 81 (29.7); 204 (25.7); 55 (20.6)
12	α-Eudesmol	28.4	1.97	222	1611	161 (99.9); 189 (96.7); 59 (85.6); 43 (74.8); 204 (73.6); 41 (72.4); 91 (53.3); 133 (49.2); 107 (48.8); 93 (48.8)
13	(-)-T-Muurolol	28.6	2.93	222	1616	49 (99.9); 161 (49.4); 95 (46.7); 41 (43.9); 204 (35.5); 121 (33.3); 79 (30.4); 105 (25.4); 81 (23.0); 55 (21.0)
14	β-Eudesmol	28.8	2.52	222	1622	59 (99.9); 149 (42.0); 43 (38.2); 41 (37.4); 108 (23.9); 93 (23.6); 79 (23.0); 81 (22.3); 67 (21.8); 164 (20.2)
15	α-Cadinol	28.9	8.34	222	1623	43 (99.9); 95 (62.7); 121 (46.8); 41 (44.0); 204 (28.5); 79 (27.7); 81 (25.6); 161 (24.1); 93 (23.3); 105 (23.1)

^a^ RT, retention time; ^b^ RP, retention percentage; ^c^ RI, retention index.

**Table 2:** Effective concentration (EC_50_) values of *C. citratus* EO, citronellal, and nerol against mycelial growth of *Botrytis cinerea* after 3-day incubation at 24 ± 1 °C.

Compound	Methods	Regression Equation	R^2^	EC_50_ (µg/mL)	95% Confidence Limits (µg/mL)
*C. citratus* EO	DC	y = 1.047x + 3.082	0.613	67.96	66.39~69.57
	VC	y = 3.874x + 0.480	0.931	14.69	13.12~16.43
Citronellal	DC	y = 0.686x + 3.733	0.721	70.32	66.81~74.02
	VC	y = 4.019x + 3.218	0.975	2.78	0.33~23.20
Nerol	DC	y = 1.357x + 1.975	0.568	169.60	163.10~176.36
	VC	y = 1.193x + 3.004	0.912	47.14	46.06~48.25

DC, direct contact assay; VC, vapor contact assay.

**Table 3:** The minimal inhibitory concentrations (MICs) and minimal fungicidal concentrations (MFCs) of *C. citratus* EO and two of its individual compounds against the spore germination of *B. cinerea* were determined after a 48 h incubation period at 24 ± 1 °C.

Compound	DC Assay		VC Assay	
	MIC (µg/mL)	MFC (µg/mL)	MIC (µg/mL)	MFC (µg/mL)
*C. citratus* EO	NI ^a^	NI	7.81	15.63
Citronellal	NI	NI	1.98	3.91
Nerol	NI	NI	15.63	31.25

^a^ NI, no inhibition at tested concentrations (≤250 µg/mL).

**Table 4:** Inhibitory effects of *C. citratus* EO and its main components on spore germination of rubber tree powdery mildew.

Compound	Regression Equation	R^2^	EC_50_ (µg/mL)	95% Confidence Limits (µg/mL)
*C. citratus* EO	y = 4.84x − 2.6	0.998	3.19	2.807~3.524
Citronellal	y = 2.06x + 0.29	0.996	0.68	0.607~0.741

## Data Availability

The original contributions presented in this study are included in the article/Supplementary Material. Further inquiries can be directed to the corresponding authors.

## Supplementary Materials

The following supporting information can be downloaded at: https://www.mdpi.com/article/10.3390/foods15030583/s1.
