# Supplementary material for: Multi-Target Antifungal Mechanism of Vapor-Phase Cymbopogon citratus Essential Oil: Effective Control of Postharvest Botrytis cinerea and Powdery Mildew
Source: Foods. 2026 Feb 5;15(3):583. doi: 10.3390/foods15030583 (PMC12896534; doi:10.3390/foods15030583)
Supplement: Supplementary file 1 [file foods-15-00583-s001.zip › Supplementary Methods.pdf]

2.2. Extraction and analysis of essential oil

The detailed methodology is as follows: GC–MS Analysis: Analysis was performed using an Agilent 7890 gas chromatograph coupled with a quadrupole mass spectrometer (Agilent 5975 N, USA). Separation was achieved on an HP-5MS fused-silica capillary column (30 m × 0.25 mm i.d., 0.25 μm film thickness; J&W Scientific, USA). The oven temperature program was set as follows: 40 °C held for 2 min, then increased at 5 °C/min to 250 °C and held for 6 min. The mass spectrometer was operated in electron-ionization (EI) mode at 70 eV, with an ion-source temperature of 230 °C. Mass spectra were acquired in full-scan mode over the range 50–550 m/z. The quadrupole temperature was 150 °C and the interface temperature was 280 °C. Helium was used as the carrier gas at a constant flow rate of 1.0 $mL \cdot min^{-1}$. Injections were performed in split mode with a split ratio of 10:1. Relative contents of identified compounds were calculated based on peak areas from the total-ion-current chromatogram. GC-FID Analysis: An Agilent 7890 gas chromatograph equipped with a flame-ionization detector (FID) was employed. The FID temperature was set at 260 °C, while the injector and oven conditions were identical to those used for GC–MS analysis. Nitrogen served as the carrier gas at a flow rate of 1.0 mL/min, and injections were likewise made in split mode (10:1).

2.4.5 Supplementary Methods: Enzymatic Activity Assays

Extract Preparation

Botrytis cinerea mycelia (0.5 g) were homogenized in 1 mL ice-cold PBS (pH 7.0), diluted to 5 mL with PBS, and centrifuged (10,000 ×g, 15 min, 4°C). The supernatant (crude enzyme extract) was used for assays. Protein concentration was determined by Bradford method (BSA standard).

1. Superoxide Dismutase (SOD) Activity

Kit: Solarbio BC0170 (Beijing, China)

Principle: SOD inhibits NBT reduction by superoxide radicals under light.

Procedure:

Table 1

Agent	Dosage
50 mM PBS (pH 7.8)	1.33 mL
130 mM Methionine	75 μL
750 μM NBT	30 μL
100 μM EDTA-Na_2	30 μL
20 μM Riboflavin	30 μL
Enzyme extract	10 μL
Reaction mix	1.5 mL

Control: Replace enzyme with PBS. Illuminate at 4,000 lux for 20 min. Measure absorbance at 560 nm.Calculation:

SOD activity

SOD activity=$\frac{(Ack-Asample)\times Vl}{0.5\times A_{Control}\times Protein\times t}\times$Dilution factor

(1 U = 50% inhibition of NBT reduction; activity in U/mg protein)

2. Catalase (CAT) Activity

Kit: Solarbio BC0200

Principle: CAT decomposes H_2O_2; activity measured by absorbance decrease at 240 nm.

Procedure:

Table 2

Agent	Dosage
50 mM PBS (pH 7.8)	850 μL
30 mM H_2O_2	100 μL
Enzyme extract	50 μL
Reaction mix	1.0 mL

Immediately record ΔA_{240}/min. Calculation:

CAT activity=$\frac{\Delta A240/min\times V}{\varepsilon\times d\times m}\times 1000$

(ε = 0.0436 mM^{-1} cm^{-1}; 1 U = decomposition of 1 μmol H_2O_2/min; activity in U/mg protein)

3. Peroxidase (POD) Activity

Kit: Solarbio BC0090

Principle: POD oxidizes guaiacol to tetraguaiacol; monitored at 470 nm.

Procedure:

Table 3

Agent	Dosage
50 mM PBS (pH 7.8)	2.75 mL
2% (v/v) Guaiacol	50 μL
30 mM H_2O_2	100 μL
Enzyme extract	100 μL
Reaction mix	3.0 mL

Record ΔA_{470}/min. Calculation:

POD activity=$\frac{\Delta A470/min \times V}{\varepsilon \times d \times m} \times 1000$

(ε = 26.6 mM^{-1} cm^{-1}; 1 U = formation of 1 μmol tetraguaiacol/min; activity in U/mg protein)

4. Succinate Dehydrogenase (SDH) Activity

Kit: Solarbio BC0950

Principle: SDH reduces succinate; MTT is reduced to formazan (measured at 600 nm).

Procedure:

Prepare working solution by mixing reagents per kit instructions.

Table 4

Agent	Dosage
Working solution	2.6 mL
Enzyme extract	100 μL
Reaction mix	2.7 mL

Incubate at 37°C. Measure A_{600} at T = 5 s (A_1) and T = 65 s (A_2). Calculation:

SDH activity=$\frac{(AT1 - AT2) \times V}{\varepsilon \times d \times m} \times 1000$

(ε = 17 mM^{-1} cm^{-1}; 1 U = oxidation of 1 nmol succinate/min; activity in U/mg protein).

5 Key Notes for Reproducibility

5.1 Replicates: All assays performed in triplicate.

5.2 Controls: Included enzyme-free and substrate-free blanks.

5.3 Units: Specific activity expressed as U/mg protein (protein determined by Bradford assay).

5.4 Kit Validation: Protocols strictly followed manufacturer specifications (Solarbio®).

6 Critical Steps:

SOD: Reactions conducted under uniform light intensity.

CAT/H_2O_2: Freshly prepared and kept in dark.

SDH: Precisely timed readings within 60 s.

This supplementary section provides complete methodological transparency while maintaining brevity in the main text. For kit verification, catalog numbers are: BC0170 (SOD), BC0200 (CAT), BC0090 (POD), BC0950 (SDH).
